# A commentary on ‘Effects of machine perfusion strategies on different donor types in liver transplantation: a systematic review and meta-analysis’

**DOI:** 10.1097/JS9.0000000000000730

**Published:** 2023-09-13

**Authors:** Gang Tang, Linyu Zhang, Rongxing Zhou

**Affiliations:** a Department of General Surgery, Division of Biliary Tract Surgery, West China Hospital; b Center for Translational Medicine, West China Second University Hospital, Sichuan University, Chengdu, Sichuan, People’s Republic of China

*Dear Editor*,

With great interest, we read the article entitled ‘Effects of machine perfusion strategies on different donor types in liver transplantation: a systematic review and meta-analysis’ recently published in the *International Journal of Surgery* by Liang *et al*.^1^. The authors evaluated the benefits of different machine perfusion strategies for different donor types through a systematic review and meta-analysis. They found that compared with static cold storage (SCS), hypothermic machine perfusion (HMP) significantly reduced the risk of non-anastomotic biliary stricture, early allograft dysfunction, major complications, primary non-function, post-reperfusion syndrome, acute cellular rejection, and improved one-year graft/recipient outcomes. Normothermic machine perfusion (NMP) has a certain auxiliary effect on the efficacy of extended criteria donor (ECD) liver transplantation, and normothermic regional perfusion (NRP) can significantly improve the prognosis of donation after circulatory death (DCD) liver transplantation recipients. We would like to congratulate the authors for writing such a rich and novel article on this topic. However, we believe that the veracity of some of the conclusions raises questions.

First, a serious flaw in the statistical methods is worth pointing out. The authors included 9 randomized controlled trials and 30 cohort studies, and estimated effect measures were calculated for event-related outcomes as odds ratio (OR). According to the *Cochrane Handbook for Systematic Review*, when the incidence of an outcome of interest in the study population is low, such as 10%, the OR is close to the risk ratio (RR). However, when the incidence of an outcome of interest is high (>10%), the OR will overestimate or underestimate the RR, and this overestimation or underestimation becomes larger with increasing incidence of the outcome^2^. In this study, the major complication rate, one-year graft survival rate, and one-year recipient survival rate were 34%, 89%, and 91%, respectively (Fig. 2), and therefore RR should be adopted. However, the authors used OR as the effect size, which may seriously undermine the reliability and validity of the study results.

Secondly, we are very skeptical about the authenticity of some of the extracted data. In the study by Rayar *et al*.^3^, 1-year graft survival and 1-year patient survival in the SCS group were 89.5% (59/69) and 91.3% (63/69), respectively. However, in the forest plot of Liang *et al*., the 1-year graft survival and 1-year patient survival in the SCS group (Rayar *et al*., 2021) were 62 (Fig. 2D) and 43 (Fig. 2E), respectively. The inaccuracy of the extracted data may affect the authenticity of the results of meta-analysis. In addition, the authors defined major complications as Clavien–Dindo (CD) grade ≥3, whereas the study by Ravaioli *et al*.^4^ only provided data on CD ≥3b. The authors’ direct inclusion of these data for analysis may compromise the validity of the pooled results. Therefore, we reanalyzed the results of this meta-analysis using updated data and with RR as the effect size. Most of the reanalysis results were consistent with the authors. However, some key indicators are inconsistent with the results of the authors. For overall effects on all donor types, HMP did not improve major complications (RR 0.79, 95% CI 0.60–1.03, *P*=0.08) (Fig. 1A) and one-year patient survival (RR 1.03, 95% CI 1.00–1.06, *P*=0.09) (Fig. 1B), compared to SCS. Similarly, there was no significant difference in the major complications (RR 0.71, 95% CI 0.46–1.11, *P*=0.14) (Fig. 1C) and one-year patient survival rates (RR 1.03, 95% CI 1.00–1.07, *P*=0.07) (Fig. 1D) between the HMP and the SCS group in the ECD liver transplantation. Therefore, the benefit of HMP on major complications and patient survival needs to be treated with caution.

**Figure 1:**
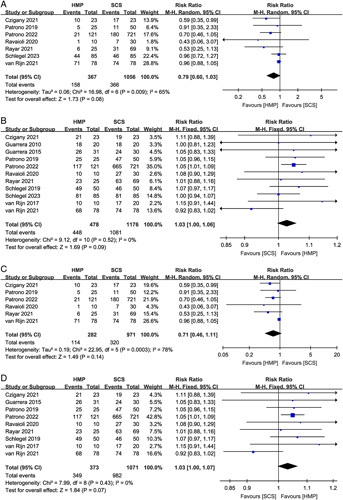
Forest plot of (A) major complications (all donor types), (B) one-year patient survival (all donor types), (C) major complications (extended criteria donor – ECD), and (D) one-year patient survival (ECD) between HMP and SCS. HMP, hypothermic machine perfusion; SCS, static cold storage.

Finally, the comprehensive inclusion of eligible evidence is a key element of systematic reviews. However, we found that a study involving 280 patients was published in 2020 by Mueller *et al*.^5^, which evaluated the application of HMP in liver transplantation for hepatic carcinoma, was not included in this meta-analysis. The authors need to clarify why this study was not included since omitting eligible studies increases the risk of bias.

In conclusion, we highly appreciate the authors’ efforts in summarizing the evidence on the effects of machine perfusion on liver transplantation. However, appropriate statistical methods and careful analysis of the data presented may have enhanced the robustness and accuracy of the findings and enhanced the clinical impact of this study.

## Ethical approval

Not applicable.

## Consent

This study did not involve patients or volunteers, and consent was not required in this study.

## Sources of funding

Not applicable.

## Author contribution

G.T. and L.Z.: data analysis; and writing study design.

## Conflicts of interest disclosure

There are no conflicts of interest.

## Research registration unique identifying number (UIN)

Not applicable.

## Guarantor

Rongxing Zhou.

## Data availability statement

The raw data were all collected in the included studies. We declare the authenticity of the data.

## Provenance and peer review

Commentary, internally reviewed.
